# Genetic risk factors in Finnish patients with Fuchs endothelial corneal dystrophy

**DOI:** 10.1111/aos.70144

**Published:** 2026-04-20

**Authors:** Inka‐Tuulevi Vähämäki, Annamari T. Immonen, Joel T. Rämö, Aino M. Jaakkola, Kari Krootila, Maria Kaukonen, FinnGen, Aarno Palotie, Hannes Lohi, Tero T. Kivelä, Joni A. Turunen

**Affiliations:** ^1^ Eye Genetics Group Folkhälsan Research Center, Biomedicum Helsinki Helsinki Finland; ^2^ Canine Genetics Group Folkhälsan Research Center, Biomedicum Helsinki Helsinki Finland; ^3^ Department of Medical and Clinical Genetics, Faculty of Medicine University of Helsinki Helsinki Finland; ^4^ Department of Veterinary Biosciences, Faculty of Veterinary Medicine University of Helsinki Helsinki Finland; ^5^ Department of Ophthalmology University of Helsinki, and Helsinki University Hospital Helsinki Finland; ^6^ Institute for Molecular Medicine Finland (FIMM), Helsinki, Institute of Life Science (HiLIFE) University of Helsinki Helsinki Finland; ^7^ Broad Institute of MIT and Harvard Cambridge Massachusetts USA; ^8^ Massachusetts Eye and Ear Boston Massachusetts USA; ^9^ Psychiatric and Neurodevelopmental Genetics Unit Massachusetts General Hospital and Harvard Medical School Boston Massachusetts USA; ^10^ Analytic and Translational Genetics Unit Massachusetts General Hospital and Harvard Medical School Boston Massachusetts USA; ^11^ Department of Neurology Massachusetts General Hospital Boston Massachusetts USA; ^12^ Stanley Center for Psychiatric Research Broad Institute of MIT and Harvard Cambridge Massachusetts USA; ^13^ Department of Pathology HUSLAB Helsinki Finland

**Keywords:** AGBL1, FECD, Fuchs corneal dystrophy, repeat expansion, TCF4

## Abstract

**Purpose:**

To study the genetic risk factors of Fuchs endothelial corneal dystrophy (FECD) in the Finnish population using hospital‐based and large biobank cohorts.

**Methods:**

We genotyped a cohort of 107 Finnish patients with FECD for the primary associated genetic risk factor, the *TCF4* (CTG)_>50_ expansion, and studied their clinical phenotype. For 18 *TCF4* (CTG)_≤50_ patients, we performed exome‐based candidate gene analysis. We also utilised FinnGen biobank‐based samples to perform a genome‐wide association study (GWAS).

**Results:**

In the cohort, 83% (89/107) carried one allele with the *TCF4* (CTG)_>50_ expansion. Those without the expansion were younger at enrolment and at the time of first keratoplasty (*p* = 0.011, *p* = 0.044, respectively). A genome‐wide association study of 892 patients and 497 827 controls identified a lead variant rs11659764 (AF = 2.3%, OR = 8.2, *p* = 2.9 × 10^−195^). Genotyping showed that 89% of *TCF4* (CTG)_>50_ carriers had the associated TA genotype rather than the TT genotype, whereas none of the *TCF4* (CTG)_≤50_ carriers did. Exome‐based analyses of the 18 *TCF4* (CTG)_≤50_ carriers identified a candidate gene variant, *AGBL1* c.901 + 2 T>G in one patient.

**Conclusion:**

In Finland, the *TCF4* (CTG)_>50_ expansion is a significant risk factor for FECD. A nearby single‐nucleotide polymorphism (SNP) was found to perform well as a surrogate for genotyping. However, not all patients carried the expanded repeat, and a variant in a previously associated gene was observed in a single patient. Further research is needed to investigate the origins of FECD in individuals without the repeat expansion.

## 1 INTRODUCTION

Fuchs endothelial corneal dystrophy (FECD, MIM 136800) causes a bilateral gradual loss of corneal endothelial cells, affecting cell morphology and function, resulting in characteristic guttae and thickening of Descemet's membrane (DM). As the disease progresses, structural changes of the stroma, stromal and epithelial edema along with subepithelial fibrosis and scarring are often observed (Adamis et al., 1993; Ong Tone et al., 2021; Weiss et al., 2024). Typically diagnosed in individuals over 40 years of age, patients with FECD commonly experience reduced visual acuity, reduced contrast sensitivity and photophobia (Watanabe et al., 2015; Weiss et al., 2024). Notably, females exhibit a higher susceptibility to FECD, with approximately twofold higher likelihood of being affected compared to males (Aiello et al., 2022; De Piano et al., 2024; Liu et al., 2020; Nakagawa et al., 2023; Ong Tone et al., 2021).

Due to the lack of effective treatment options for cell loss in FECD, corneal transplantation, keratoplasty, has become the main approach to restore visual acuity, making FECD a leading cause of corneal transplantations globally, particularly after the introduction of posterior lamellar keratoplasty techniques (Gain et al., 2016; Touirssa et al., 2025). The two most performed transplantation methods include Descemet stripping automated endothelial keratoplasty (DSAEK), involving the replacement of the affected endothelium and DM by donor endothelium, DM, and a thin layer of stroma. A more advanced and surgically intricate procedure is Descemet membrane endothelial keratoplasty (DMEK), where both the host endothelium and DM are replaced with corresponding donor tissue (Blitzer & Colby, 2020). Both methods contribute to visual acuity improvement, with DMEK demonstrating faster recovery and a reduced risk of graft rejection (Blitzer & Colby, 2020; Price et al., 2009). However, the scarcity of donor corneas has prompted the exploration of alternative treatment approaches, such as Descemet stripping only (DSO) and the investigation of topical Rho‐associated kinase (ROCK) inhibitors (Borkar et al., 2016; Davies et al., 2021; Gain et al., 2016; Moloney et al., 2017). Furthermore, emerging cell‐based therapies hold the potential to transform the treatment of FECD. A landmark clinical trial in Japan demonstrated that an intracameral injection of allogeneic cultured corneal endothelial cells is both safe and effective, ultimately leading to regulatory approval (Kinoshita et al., 2018, 2025).

The aetiology of FECD is multifactorial, involving environmental factors, increased oxidative damage and a complex genetic architecture (Azizi et al., 2011; Jurkunas et al., 2010; Ong Tone et al., 2021). Early‐onset FECD, manifesting in the first to third decade of life with a distinct phenotype, is linked to an autosomal dominant variant in the collagen type VIII alpha 2 chain gene (*COL8A2*, MIM 136800) (Biswas et al., 2001; Gottsch et al., 2005). Furthermore, rare variants have also been implicated in the transcription factor 4 (*TCF4*, MIM 602272), solute carrier family 4 member 11 (*SLC4A11*, MIM 610206), zinc finger E‐box binding homeobox 1 (*ZEB1*, MIM 189909), AGBL carboxypeptidase 1 (*AGBL1*, MIM 615496), lipoxygenase homology PLAT domains 1 (*LOXHD1*) and TSPO associated protein 1 (*TSPOAP1*) genes (Baratz et al., 2010; Bhattacharyya et al., 2024; Mehta et al., 2008; Nanda & Alone, 2019; Riazuddin et al., 2012, 2013; Vithana et al., 2008; Wieben et al., 2012, 2018). Recently, variants in the microRNA 184 (*MIR184*) were identified, expanding the genetic background of FECD to noncoding variants (Abreu Costa et al., 2025). Nevertheless, the presumed disease‐causing variants within these genes contribute to only a minor portion of total FECD.

Additional genome‐wide associated loci related to FECD have highlighted significant associations within chromosome 18, particularly in the genomic region encompassing the *TCF4* gene (Afshari et al., 2017; Baratz et al., 2010). Other significantly associated loci in additional genomic regions include, for example, KN motif and ankyrin repeat domains 4 (*KANK4*), laminin subunit gamma 1 (*LAMC1*), laminin subunit beta 1 (*LAMB1*), long intergenic non‐protein‐coding RNA 970/ATPase Na+/K+ transporting subunit beta 1 (*LINC00970*/*ATP1B1*), single‐stranded DNA binding protein 3 (*SSBP3*), thrombospondin type 1 domain containing 7A (*THSD7A*), P53‐induced death domain protein 1 (*PIDD1*), RAR related orphan receptor A (*RORA*), heparan sulphate‐glucosamine 3‐sulfotransferase 3B1 (*HS3ST3B1*), laminin subunit alpha 5 (*LAMA5*) and collagen type XVIII alpha 1 chain (*COL18A1*) (Afshari et al., 2017; Gorman et al., 2024). However, only one pathogenic variant has been identified in *LAMC1* to date (Gorman et al., 2024; Wieben et al., 2018). Adding further to the genetic heterogeneity of FECD, a recent GWAS identified multiple novel loci including semaphorin 6A (*SEMA6A*), DnaJ heat shock protein family (Hsp40) member C19 pseudogene 3 (*DNAJC19P3*) and long non‐protein‐coding RNAs (lncRNAs) (Fechner et al., 2025).

The primary genetic risk factor contributing to FECD is a *TCF4* (18q21.2) intronic (CTG)_n_ repeat expansion. The expansion frequency varies between ancestries and is observed at the highest frequencies in populations of European ancestry (Eghrari et al., 2017; Fautsch et al., 2021; Nakano et al., 2015; Rao et al., 2017; Wieben et al., 2012; Zhang et al., 2025). *TCF4*, a member of the basic helix–loop–helix family of transcription factors, plays important roles in development and transcriptional regulation. The *TCF4* (CTG)_n_ triplet repeat expansion is hypothesised to cause dysregulation of its transcripts in an isoform‐specific pattern, potentially resulting in changes in its expression in a length‐dependent manner (Bhattacharyya et al., 2024; Fautsch et al., 2021; Nakagawa et al., 2023; Sirp et al., 2020). One suggested disruption is caused by an accumulation of nuclear RNA foci, which sequester mRNA‐splicing factors, *MBNL1* and *MBNL2*, leading to abnormal splicing events (Du et al., 2015; Mootha et al., 2015; Wieben et al., 2018; Zarouchlioti et al., 2018). Recently, other splicing factors have also been proposed to play a role in RNA toxicity (Bhattacharyya et al., 2024). Other proposed methods include toxic repeat‐associated non‐ATG translation, dysregulation of ion transport and somatic instability (Fautsch et al., 2021; Gorman et al., 2024; Hafford‐Tear et al., 2019; Soragni et al., 2018; Zarouchlioti et al., 2024).

Here, we describe genetic risk factors underlying FECD in Finnish patients. First, we studied the prevalence of the well‐established risk factor *TCF4* triplet (CTG)_>50_ repeat expansion in a cohort of 107 patients. Secondly, we performed candidate gene analysis in *TCF4* (CTG)_≤50_ patients to identify alternative genetic risk factors. To further explore genetic associations, we conducted a biobank‐based genome‐wide association study (GWAS) including 892 patients and 497 827 controls.

## 2 METHODS

### 2.1 Patients in the Helsinki University Hospital cohort

Patients were eligible for inclusion in this study if diagnosed with FECD (ICD‐10 H18.51) between January 1, 2019, and July 31, 2022, at the Corneal and Anterior Segment Surgery Service, Helsinki University Hospital. In total, 226 patients were invited, of whom 113 chose to participate. Six patients were excluded from the final cohort due to previous ocular trauma, prior laser‐assisted in situ keratomileusis (LASIK) procedures or corneal injury from cataract surgery. The final cohort comprised 107 patients. Samples from 202 controls were included from the Finnish Red Cross Biobank. All control samples were from males, and no phenotype information was available for this cohort. The study was approved by the Institutional Review Board of the Hospital Region of Helsinki and Uusimaa and adhered to the principles outlined in the Declaration of Helsinki. Written consent was obtained from all participants.

All participating patients had undergone clinical ophthalmological examination, and clinical data collected from their medical records included clinical characteristics, best‐corrected visual acuity (BCVA, Snellen), anterior segment optical coherence tomography (AS‐OCT; CASIA2, Tomey, Nagoya, Japan) and specular microscopy (EM‐4000, Tomey, Nagoya, Japan).

### 2.2 Genetic analysis of the *TCF4* (CTG)_n_ triplet repeat sequence and rs11659764

A peripheral blood sample was collected from 113 participating patients. Genomic DNA was extracted from the white blood cells using the semi‐automated Chemagen extraction robot (PerkinElmer Chemagen Technologie GmbH, Baeswieler, Germany). Subsequently, DNA concentration and purity were assessed using a Nanodrop ND‐1000 UV/Vis Spectrophotometer (Nanodrop Technologies, Wilmington, DE, USA) following the manufacturer's instructions.

All patient and control samples were subjected to *TCF4* (CTG)_n_ repeat sequence genotyping through short tandem repeat analysis. Both traditional polymerase chain reaction (PCR) and triplet‐repeat primed PCR were performed. Primer sequences for the PCRs were obtained from the studies of Wieben et al. (2012) for standard PCR, and Vasanth et al. (2015) adapted from Mootha et al. (2014) for the triplet repeat primed PCR to detect the longer expanded allele lengths (Mootha et al., 2014; Vasanth et al., 2015; Wieben et al., 2012). All PCR products were amplified using Biotools DNA Polymerase (Biotools B&M Labs, Valle de Tobalina, Madrid, Spain). Fragment analysis was performed by capillary electrophoresis by an ABI3730xl DNA analyser (Applied Biosystems, Thermo Fisher Scientific, Waltham, MA, USA) at the Institute for Molecular Medicine Finland (FIMM; Helsinki, Finland). The fragment data were analysed using Applied Biosystems (Thermo Fisher Scientific, Waltham, MA, USA) Microsatellite Analysis (version 1.2‐PRC‐build08) and Peak Scanner (version 3.1.1‐PRC‐build07) applications. The expanded allele limit was set at >50 CTG repeats.

For rs11659764 SNP genotyping, primers (F) TCTGCTGTTCAAAAGACACCA and (R) TCCCATTTGCTCCACATCCT were designed in‐house using Primer3 (Kõressaar et al., 2018). PCR products were Sanger sequenced at FIMM, and sequences analysed with UGene (Okonechnikov et al., 2012). Primers used for genotyping *GRHL2* and *OVOL2* promoter variants were (F) CTGCTGCTACTACTACCACTTTG and (R) GGAGGAAGGCGGGATGTC, and (F) TCGAGGTGCAGGTGATGG and (R) CTCATCCCAGCTGCGGAC, respectively.

### 2.3 Exome sequencing and candidate gene analysis

Eighteen patients without the *TCF4* (CTG)_>50_ genotype were chosen for exome sequencing (ES). The sequencing and variant calling were performed by Novogene, Cambridge, UK. The exome libraries were prepared using SureSelect Human All Exon V6 method (Agilent Technologies, Santa Clara, CA, USA), and the amplified fragments were sequenced on the Illumina platform employing the PE150 strategy (Illumina, San Diego, CA, USA). The bioinformatic analysis included data quality control of FASTQ files to discard paired‐end reads containing adapter contamination, more than 10% of uncertain bases in either one read and reads that have >50% proportion of low‐quality (Phred <5) bases. Next, sequence alignment to human hg38 was performed using Burrows–Wheeler Aligner (v0.7.17, Li & Durbin, 2009), Sambamba (v0.7.1, Tarasov et al., 2015) for BAM file sorting and Picard Toolkit (v2.18.9, Broad Institute, GitHub Repository) to mark duplicate reads. Single nucleotide variants (SNVs) and insertions and deletions (INDELs) were detected using GATK (V.4.0, DePristo et al., 2011), and annotation of the variants was done using ANNOVAR (Wang et al., 2010).

For variant filtering, a candidate gene approach was chosen, focusing on genes previously associated with Fuchs' dystrophy and posterior polymorphous corneal dystrophy (PPCD). The selected genes included: *AGBL1, COL8A2, GRHL2, LOXHD1, OVOL2*, *SLC4A11*, *TCF4*, *TSPOAP1, ZEB1*, and associated loci in *COL18A1*, *HS3ST3B1*, *KANK4*, *LAMA5*, *LAMB1*, *LAMC1*, *LINC00970/ATP1B1*, *PIDD1*, *RORA*, *SSBP3* and *THSD7A*. The *ATP1B1* gene, identified as a secondary associated locus in the FinnGen biobank genome‐wide association study, was included in the analysis. Exonic and splicing variants within candidate genes with a minor allele frequency (AF) of ≤0.1 in the Finnish population according to the gnomAD database (Karczewski et al., 2020) were included. Additionally, Sanger sequencing was performed for seven PPCD‐associated promoter region variants in *GRHL2* (c.20 + 133delA, c.20 + 257delT, c.20 + 544G>T) and *OVOL2* (c. −61G>A, c. −274 T>G, c. −307 T>C, c. −370 T>C). The pathogenicity of the variants was evaluated using the guidelines by the American College of Medical Genetics and Genomics (ACMG), implemented in VarSome (Release 11.8) (Kopanos et al., 2019; Richards et al., 2015). SpliceAI was used to predict pathogenicity of splice site variants (Jaganathan et al., 2019).

### 2.4 FinnGen cohort description

FinnGen is a collection of prospective Finnish epidemiological and disease‐based cohorts and hospital biobank samples (https://www.finngen.fi/en) (Kurki et al., 2023). The FinnGen data used in this study comprises 500 348 individuals from FinnGen Data Freeze 12.

FinnGen participants were genotyped using ThermoFisher, Illumina and Affymetrix arrays. Samples that were not of genetically inferred Finnish ancestry or that were twins or duplicates of included samples were not included in the study. Genotype imputation was performed using a population‐specific SISu v4.2 imputation reference panel comprised of 8557 whole genomes and the Beagle v4.1 software as detailed in a publicly available protocol (https://doi.org/10.17504/protocols.io.xbgfijw).

Data for all individuals were linked by unique national personal identification numbers to the national hospital discharge registry (available 1969–), specialist outpatient visit registry (1998–) and primary health care outpatient visit registry (2011–). We first excluded all participants with at least one diagnosis of keratoconus (H18.6) or amyloidosis (E85). We then assigned case status to all participants with a first diagnosis of H18.5 after age 50. All remaining participants were assigned control status.

### 2.5 Genome‐wide association studies in FinnGen

We conducted a case–control genome‐wide association study of FECD in FinnGen with REGENIE v2.2.4, adjusting for sex, age at end of follow‐up or death, the first ten principal components of genetic ancestry, genotyping array and genotyping batches. We excluded results for variants with INFO scores <0.6 or minor allele frequencies <0.1%.

### 2.6 Statistical analysis

Correlation between patient clinical characteristics and *TCF4* (CTG)_n_ repeat expansion length was analysed with Spearman's rank correlation coefficient. For analysis, the mean value of both eyes was used when available, and a single value when only one eye was analysed. First‐surgery analysis refers to the eye that was operated on first. If the fellow eye later underwent surgery, analyses for that eye are referred to as second‐surgery analyses. Fisher's exact test was used to test the association between rs11659764 and *TCF4* (CTG)_≤50_ and *TCF4* (CTG)_>50_ groups. Additionally, comparison of clinical characteristics between *TCF4* (CTG)_≤50_ and *TCF4* (CTG)_>50_ groups was done using an independent *t*‐test for equal and unequal variance and the Mann–Whitney *U* test. Multiple linear regression was used to assess the predictive value of age at enrolment, *TCF4* (CTG)_n_, and whether cataract extraction had been performed prior to central corneal thickness (CCT) measurement on the preoperative CCT (SM). Right and left eyes were analysed separately. All statistical analyses were performed using IBM SPSS versions 27.0, 29.0.1.0, 29.0.2.0 and 31.0.1.0 (IBM Corp, Armonk, NY, USA), with a significance threshold set at *p*‐values ≤0.05.

## 3 RESULTS

The study included 107 patients diagnosed with FECD, of whom 77 (72%) were females and 30 (28%) were males. The median age of the patients at the time of study enrolment was 69.8 years (interquartile range [IQR] 64–75). The majority of the patients had undergone at least one keratoplasty, with an overall transplantation rate of 79% for the included eyes (169/214) (Table 1). Among the keratoplasties, DMEK was the most common method, accounting for 59% (100/169) of all procedures, followed by DSAEK at 37% (62/169) and penetrating keratoplasty (PKP) at 4% (7/169).

**TABLE 1:** Characteristics of the patients enrolled in the study. The 107 patients included in the study were divided into *TCF4* (CTG)_>50_ and *TCF4* (CTG)_≤50_ groups for comparison of genetic and clinical characteristics. The mean value of both eyes was used when available, and a single value was used when only one eye had been analysed.

	All	*TCF4* (CTG)_>50_	*TCF4* (CTG)_≤50_
Patients, *N*	107	89	18
Female‐to‐male ratio	2.6	2.3	5.0
Age at study enrolment, mean (SD)	69.8 (8.5)	70.7 (7.8)	65.2 (10.7)
CCT^a^ (AS‐OCT), μm, median (IQR, *N*)	597 (565–625, 96)	604 (576–634, 78)	565 (540–603, 18)
CCT^a^ (SM), μm, median (IQR, *N*)	608 (575–633, 103)	614 (580–639, 86)	575 (541–606, 17)
BCVA (decimal), median (IQR, *N*)	0.4 (0.3–0.5, 107)	0.4 (0.3–0.5, 89)	0.5 (0.4–0.6, 18)
BCVA (logMAR), median (IQR, *N*)	0.4 (0.3–0.6, 107)	0.4 (0.3–0.6, 89)	0.3 (0.2–0.4, 18)
BCVA (decimal), better eye, median (IQR, *N*)	0.5 (0.32–0.63, 107)	0.5 (0.32–0.63, 89)	0.5 (0.4–0.8, 18)
BCVA (logMAR), better eye, median (IQR, *N*)	0.3 (0.2–0.49, 107)	0.3 (0.20–0.49, 89)	0.3 (0.1–0.4, 18)
Pre‐keratoplasty cataract extraction^b^, *N* eyes (%)	43 (20)	38 (21)	5 (14)
Keratoplasty, *N* one eye/both eyes (all eyes, %)	35/67 (169, 79)	28/58 (144, 81)	7/9 (25, 69)
Age at keratoplasty, years, median (IQR, *N*)	70 (63–74, 102)	71 (63–74, 86)	64 (56–74, 16)
Rs11659764 genotypes (TA/TT)	80/27	80/9	0/18

Abbreviations: AS‐OCT, anterior segment optical coherence tomography; BCVA, best‐corrected visual acuity; CCT, central corneal thickness; IQR, interquartile range; SD, standard deviation; SM, specular microscopy.

^a^ Measured at the centre of cornea; on the last visit before keratoplasty or at the last review visit.

^b^ Both eyes included.

### 3.1 *TCF4* (CTG)_n_ genotypes in the Finnish patients and control individuals

Of the 107 patients included in the study, 89 (83%) were heterozygous for the *TCF4* (CTG)_>50_ repeat expansion (Figure 1).

**FIGURE 1:**
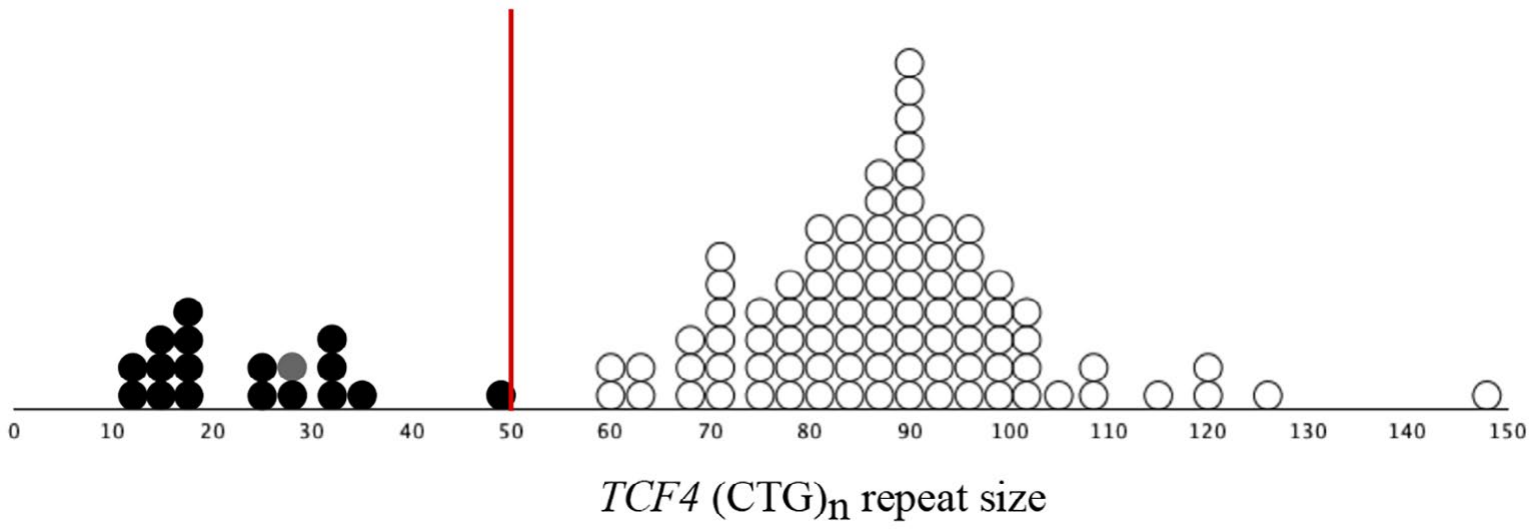
Distribution of observed *TCF4* (CTG)_n_ repeat lengths of the longer allele among 107 genotyped patients. The threshold for the expanded genotype was set at >50 CTG repeats, indicated by a red line. The average repeat length was 77 repeats: 88 in the (CTG)_>50_ (white circles), and 23 repeats in the (CTG)_≤50_ group (black circles). Patient carrying the *AGBL1* c.901 + 2 T>G variant is indicated with a grey circle. The shorter allele (not shown) had an average of 16 repeats: 17 in the (CTG)_>50_ group and 14 in the (CTG)_≤50_ group.

In the control cohort of 202 individuals, five were heterozygous for the *TCF4* (CTG)_>50_ repeat expansion, accounting for 2.5% of the cohort.

### 3.2 Genome‐wide association study in FinnGen

We identified a total of 892 patients and 497 827 controls with potential FECD based on ICD‐10 codes. In a case–control genome‐wide association study, we observed two loci at genome‐wide significance (Figure 2) and no evidence of significant inflation (genomic inflation factor = 1.02; Figure S1). In the first locus on chromosome 18, the lead variant was rs11659764 (18:55668281:T>A) located upstream of *TCF4*. Rs11659764 was carried by 20.3% of patients with potential FECD and only 2.3% of control participants (OR = 8.23, *p* = 2.9 × 10^−195^). In the second locus on chromosome 1, the lead variant was rs1200108 (1:169088731:G>A) located upstream of *ATP1B1*. Rs1200108 was carried by 57.8% of patients with potential FECD and 64.8% of control participants (OR = 0.74, *p* = 3.1 ×^−10^).

**FIGURE 2:**
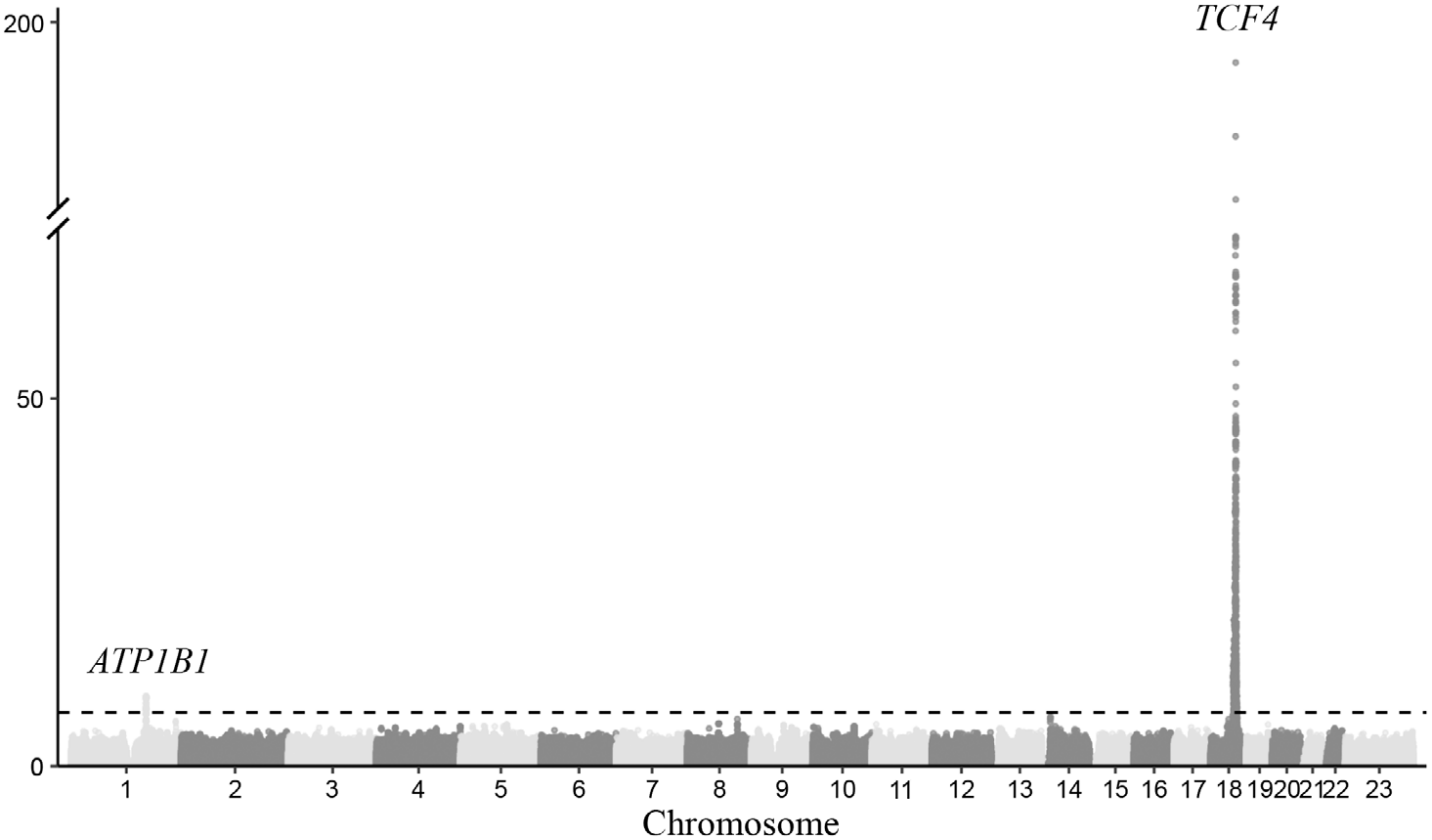
Genome‐wide association study of potential FECD in FinnGen. A total of 892 patients and 497 827 controls with potential FECD were identified based on ICD codes, and a case–control genome‐wide association study was conducted with REGENIE v2.2.4. Each genomic variant is plotted as a data point, with *p*‐values shown on the *y*‐axis on a logarithmic scale. Due to the strength of the signal on chromosome 18, the *y*‐axis includes a breakpoint to improve readability for association results in other chromosomes. The genome‐wide significance threshold (*p* = 5E‐8) is shown with a dashed line. In loci reaching genome‐wide significance, the nearest protein‐coding gene to the lead variant is labelled.

### 3.3 The lead variant rs11659764 genotypes in the patient cohort

The FinnGen GWAS lead variant, rs11659764, was genotyped in all the patients from the hospital cohort: 75% (80/107) had TA and 25% (27/107) TT genotypes. All patients with the TA genotype had the *TCF4* (CTG)_>50_ repeat expansion, and the remaining 10 patients with the expansion had the TT genotype. All the *TCF4* (CTG)_≤50_ patients had the TT genotype. The rs11659764 TA genotype was significantly associated with the *TCF4* (CTG)_>50_ expansion (Fisher's exact test, *p* = <0.001). The efficacy of the rs11659764 to indicate the presence of the *TCF4* (CTG)_>50_ repeat expansion was 89% (80/90).

### 3.4 Central corneal thickness and *TCF4* (CTG)_n_ length exhibit a correlation

Patient clinical characteristics were analysed for correlation with the *TCF4* (CTG)_n_ length. The CCT measured by specular microscopy either on the last visit before keratoplasty or at the last review visit if no surgery had been performed indicated a positive correlation with the *TCF4* (CTG)_n_ length (Spearman's rank correlation, *r*_s_ = 0.28, *n* = 103, *p* = 0.004). The CCT measured from AS‐OCT, which was taken on average 12 days later than the specular microscopy measured CCT (130/162 measurements were taken on the same date), did not exhibit a correlation with the repeat length (Spearman's rank correlation: *r*_s_ = 0.17, *n* = 96, *p* = 0.093). The observed median time lapse between both measurement methods and surgery was 90 days, and the average age of the patient at the time of the measurement was 69 years (details of ages at measurement and days between measurements can be seen in Table S1).

No correlation was observed between repeat length and age at the study enrolment date (*r*_s_ = 0.07, *n* = 107, *p* = 0.47), age at surgery (*r*_s_ = 0.026, *n* = 102, *p* = 0.79), nor first or second surgery (first surgery: *r*_s_ = 0.02, *n* = 102, *p* = 0.85, second surgery: *r*_s_ = 0.18, *n* = 67, *p* = 0.15), or BCVA in the better eye on the last visit before keratoplasty or at the last review visit if no surgery had been performed (decimal: *r*_*s*_ = −0.08, *n* = 107, *p* = 0.39; logMAR: *r*_*s*_ = 0.08, *n* = 107, *p* = 0.39).

### 3.5 *TCF4* (CTG)_≤50_ patients are younger at the time of enrolment and first keratoplasty

Comparison of the *TCF4* (CTG)_**≤**50_ and *TCF4* (CTG)_>50_ groups revealed differences in the age at enrolment (independent *t*‐test, equal variances *t*(105) = −2.6, *p* = 0.011), with a mean difference of −5.6 (95% CI, −9.8 to −1.3) (Figure 3). Similarly, the age at the first keratoplasty demonstrated a difference between the two groups (independent *t*‐test, equal variances *t*(100) = −2, *p* = 0.044), with a mean difference of −4.9 (95% CI, −9.6 to −0.14). Conversely, the difference in the ages at second keratoplasty did not reach statistical significance between the two groups (independent *t*‐test, unequal variances *t*(8.9) = −2.07, *p* = 0.069), with a mean difference of −8.3 (95% CI, −17 to 0.78), or best corrected visual acuity (BCVA) in the better eye on the last visit before keratoplasty or at the last review visit if no surgery had been performed (Mann–Whitney *U*, *U* = 607, *p* = 0.1).

**FIGURE 3:**
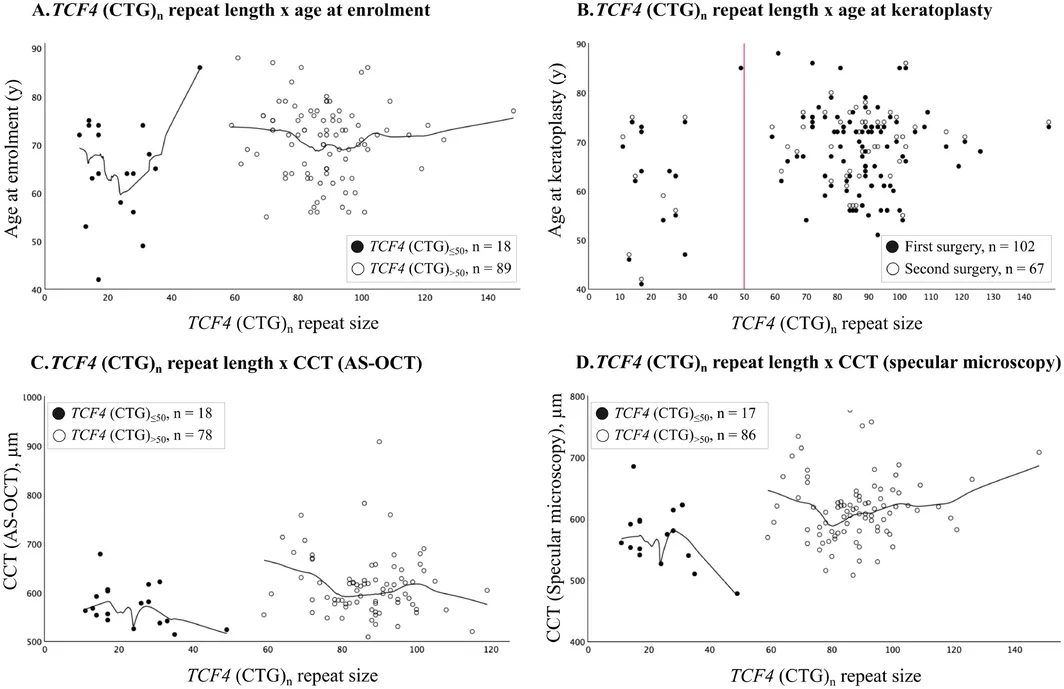
Plots representing patient clinical characteristics. Scatterplots showing age at study enrolment (a), ages at first (the eye that was operated on first) and second (if the fellow eye later underwent surgery) keratoplasty (b), and anterior segment optical coherence tomography (AS‐OCT) and specular microscopy (SM) measured central corneal thicknesses (CCT) on the last visit before keratoplasty, or at the last review visit if the patient had no corneal transplantations, plotted against the *TCF4* (CTG)_n_ repeat length. Each dot represents the mean value of both eyes when data from both eyes were available, or a single‐eye value when only one eye had been analysed. For AS‐OCT, a mean value of both eyes was available in 16/62 cases (≤50/>50) and a single‐eye value in 2/16 (≤50/>50). For SM, a mean value of both eyes was available in 16/76 cases (≤50/>50) and a single‐eye value in 1/10 (≤50/>50) (C, D). Lines represent LOESS curves (tricube smoothing) (A, C, D).

Multiple linear regression analyses were conducted for the *TCF4* (CTG)_≤50_ and *TCF4* (CTG)_>50_ groups to examine the predictors of preoperative CCT. The first model included age at enrolment and the *TCF4* (CTG)_n_ repeat length as predictors, whereas the second model also included cataract extraction status prior to CCT measurement. Both models failed to reach statistical significance in either group (Table S2).

Postoperative CCT comparisons at 3, 6, and 12 months for *TCF4* (CTG)_≤50_ and *TCF4* (CTG)_>50_ groups are provided in Table S3 owing to the small sample sizes.

### 3.6 Patient cohort with the *TCF4* (CTG)_≤50_ genotype

Among the 107 participants in the study cohort, 18 participants had a *TCF4* (CTG)_n_ length of ≤50. Fifteen patients were female (83%), and 69% (25/36) of the eyes had undergone keratoplasty. Seven patients had undergone keratoplasty in one eye and nine patients in both eyes. The ages at keratoplasty varied between 41 and 85 years (median 63 years, IQR 55–74), compared to the *TCF4* (CTG)_>50_ group, where the ages ranged from 51 to 88 (median 70 years, IQR 63–74). To assess possible variants in other FECD‐associated genes, exome sequencing (ES) was performed.

### 3.7 ES analysis suggests a potential variant in the FECD candidate gene

ES was conducted for 18 individuals with the *TCF4* (CTG)_≤50_ genotype. On average, 86 863 141 reads were collected, with a minimum of 98.5% of reads properly mapped to the reference. Exonic and splicing variants with an AF of <0.1% in the Finnish population according to the gnomAD database from FECD‐ and PPCD‐associated genes and loci were included. A total of 74 variants were included in further analysis (Table S4), comprising 72 exonic and 2 splicing variants. Among these, 73 variants were predicted to be either benign or likely benign.

A splicing variant of uncertain significance (VUS) in *AGBL1* c.901 + 2 T>G, rs138185152 (gnomAD genome AF Finnish (FIN) 0.0107 and gnomAD genome AF 0.0021), in intron 8 (NM_152336.4) of the gene is predicted to be in a donor splice site (donor loss delta score = 0.99, donor gain delta score = 0.82 (NM_152336.4)). The variant was observed in a heterozygous state in a female with a family history of FECD who underwent bilateral keratoplasty at the age of 63.

## 4 DISCUSSION

The *TCF4* (CTG)_>50_ expansion is the predominant genetic risk factor among Finnish patients with FECD, with 83% of patients carrying one copy of the expanded allele. For patients without the *TCF4* (CTG)_>50_ genotype, ES analysis identified a potential genetic risk variant in *AGBL1*, a gene previously associated with FECD. These results also suggest that additional risk factors contribute to the aetiology of FECD in patients without the expansion.

The observed *TCF4* (CTG)_>50_ genotype frequency is consistent with previously reported frequencies in populations of European ancestry (Foja et al., 2017; Viberg et al., 2022; Wieben et al., 2012). In the control group, 2,5% had the *TCF4* (CTG)_>50_ genotype. The detailed comparability of the control group to the patients is limited due to the lack of available clinical data for the control individuals. Additionally, the all‐male composition of the control group may contribute to a potentially lower frequency of observed *TCF4* (CTG)_>50_ genotypes, given the known female predominance in FECD (Aiello et al., 2022).

While assessing the correlation between the *TCF4* (CTG)_n_ repeat length and available clinical parameters, no correlations were found between age at study enrolment or age at surgery. However, a correlation emerged between CCT and the number of repeats. Notably, measurements taken with AS‐OCT did not indicate as strong an association as those measured by specular microscopy. This might indicate differences in the accuracy of one method over the other, the accuracy of manual AS‐OCT measurements or variations due to the time of day at the measurement (Fritz et al., 2019).

The severity of FECD, for example, the Krachmer grade of severity and need for keratoplasty, is known to intensify with the length of the repeat expansion (Soliman et al., 2015; Viberg et al., 2022). However, our study did not employ a classification of FECD severity beyond the CCT and patient age at enrolment or surgeries. No grading was conducted based on slit‐lamp observation of guttae, endothelial cell count, microscopy or histological findings that could provide insights into the severity and its correlation with the repeat length. Consequently, the measurement of statistical severity could not be comprehensively assessed in our study.

In a genome‐wide analysis of common variants, the most significant variant associated with inherited corneal dystrophies in our study was rs11659764, carried by 20.3% of patients with potential FECD and only 2.3% of control participants. Rs11659764 had an odds ratio (8.4) for a common variant and distinguished the *TCF4* (CTG)_>50_ cases from the non‐expanded ones with 89% accuracy. While SNP genotyping could not identify all participants with *TCF4* (CTG)_>50_, all individuals who were heterozygous for rs11659764 were *TCF4* (CTG)_>50_ cases in the studied Finnish population. Given the relative ease and increasing use of SNP genotyping within both clinical and commercial contexts, these results may inform individual risk prediction when rs11659764 is observed as an incidental finding in the general population or unaffected family members of FECD patients.

Previously, rs11659764 has been reported as the lead SNP in two studies conducted in the United States, one of which encompassed three ancestries—admixed African, European and Hispanic/Latino (Afshari et al., 2017; Gorman et al., 2024). Additionally, rs11659764 emerged as a prominent SNP in a significant locus associated with corneal resistance factor (CRF) (Khawaja et al., 2019). CRF has demonstrated a negative correlation with CCT in FECD, and it is thought to be related to corneal hydration, along with other biomechanical properties of the cornea (del Buey et al., 2009).

The initial GWAS revealed two associated loci, where a strong association of rs11659764 could possibly be attributed to a substantial proportion of patients affected by the *TCF4* (CTG)_>50_ expansion in the dataset. The second locus close to protein‐coding *ATP1B1*, with the highest associated variant rs1200108, has been previously reported as a tag SNP for another FECD‐associated risk variant rs1200114 (Afshari et al., 2017; Chakraborty et al., 2023). Rs1200108 has also been associated with corneal hysteresis, CRF and an increased risk of keratoconus in large GWAS studies (Hardcastle et al., 2021; Simcoe et al., 2020). Furthermore, the minor impact of other potential genetic risk factors contributing to FECD in Finnish patients may not provide sufficient statistical power to attain significance in the studied cohort. It is also worth noting that the cases in the dataset may include other inherited corneal dystrophies, each with a distinct genetic background. This diversity could obscure the already subtle signals of rare FECD‐associated loci within the dataset. Interestingly, other large GWAS on FECD cohorts have identified more than ten associated loci, with the *TCF4* locus on chromosome 18 having been the most significant (Afshari et al., 2017; Fechner et al., 2025; Gorman et al., 2024).

Within the cohort, 18 patients exhibited *TCF4* (CTG)_≤50_ genotypes. On average, this group was younger at the time of enrolment and at the time of their first keratoplasty compared to the *TCF4* (CTG)_>50_ group. The age disparity could potentially be attributed to an earlier onset of the disease in the *TCF4* (CTG)_≤50_ patients or the presence of more severe symptoms, resulting in more frequent clinic visits at a younger age. However, the absence of a severity classification in our study makes it challenging to definitively ascertain whether the disease in *TCF4* (CTG)_≤50_ patients differs in severity from the *TCF4* (CTG)_≤50_ group. Another point to note is that the female‐to‐male ratio in the *TCF4* (CTG)_≤50_ group was more than double to that of the *TCF4* (CTG)_>50_ group. This aligns with previous findings and might reflect hormonal factors related to menopause, as the CECs in women appear more susceptible to oestrogen effects (De Piano et al., 2024; Liu et al., 2020, 2025; Petrela et al., 2025).

One patient in the *TCF4* (CTG)_≤50_ group had 49 repeats, placing them in an intermediate range where pathogenicity remains uncertain. As 40 repeats is also used as a cutoff in literature, and intermediate repeats (40–49) are rare, the unambiguous nature of this intermediate repeat range suggests that this patient might potentially exhibit effects of the expanded triplet repeat (Viberg et al., 2022; Wieben et al., 2012).

The 18 patients in the *TCF4* (CTG)_≤50_ group were screened for variants in FECD‐associated risk genes and loci, as well as PPCD‐associated promoter variants in *GRHL2* and *OVOL2*. Within the screened genes, one variant was predicted to be of unknown significance: an *AGBL1* splicing variant observed in a single patient. *AGBL1* has been suggested as a contributing locus for late‐onset FECD, with observed interaction with *TCF4* potentially driving the pathogenesis (Riazuddin et al., 2013). However, subsequent functional studies of the gene did not find *AGBL1* to be expressed in corneal endothelial tissue samples from eyes with FECD or normal corneas, raising questions about the pathogenic nature of the variant (Frausto et al., 2014; Liu et al., 2025; Tsedilina et al., 2023; Wieben et al., 2018). Further analysis of the observed variant is needed to declare potential familial segregation and elucidate the connection with FECD.

To address the missing heritability in the *TCF4* (CTG)_≤50_ group, the next phase of the study will include an investigation of the recently identified noncoding variants and associated genetic loci (Abreu Costa et al., 2025; Fechner et al., 2025).

In summary, we found that the *TCF4* repeat expansion is the most significant genetic risk factor for FECD in Finland. However, not all patients carried the expanded repeat, and a variant within a previously linked gene potentially explained the condition in only a single patient. These findings underscore the need for further research into the genetic basis of FECD in individuals without the repeat expansion, necessitating larger sample sizes for a comprehensive investigation.

## FUNDING

This study was funded by Sigrid Juselius Foundation, Mary and Georg C. Ehrnrooth Foundation; Evald and Hilda Nissi Foundation; The Eye and Tissue Bank Foundation, Helsinki, Finland.

## Supporting information


**Figure S1**. **Quantile‐quantile plot of p‐values from a genome‐wide association study of potential FECD in FinnGen**. A total of 892 patients and 497 827 controls with potential FECD were identified based on ICD codes, and a case–control genome‐wide association study was conducted with REGENIE v2.2.4. The expected and observed *p*‐values are plotted on the *x* and *y*‐axis, respectively. The red line corresponds to a theoretical distribution with no deviation from expected *p*‐values.

**Table S1**. **Details of correlation analysis performed on TCF4 (CTG)**_**n**_ length **and patient clinical characteristics**. Spearman’s rank correlation was used for statistical analyses. Average days between central corneal thickness measurements and keratoplasties as well as patient ages at measurements were calculated using SPSS versions 27.0 and 29.0.2.0 (IBM Corp, Armonk, NY), and Microsoft Excel (V. 16.79.1, Microsoft Corporation, WA, USA).

**Table S2**. **Results of multiple linear regression analysis**. Two models were used for *TCF4* (CTG)_≤50_ and *TCF4* (CTG)_>50_ groups to examine the predictors of preoperative CCT (measured by specular microscopy). The first model included *TCF4* allele length and age at enrolment. Second model included *TCF4* allele length, age at enrolment, and cataract extraction status prior to CCT measurement. Left and right eyes were analysed separately.

**Table S3**. **Comparison of anterior segment optical coherence tomography (AS‐OCT) measured central corneal thickness (CCT) between TCF4 (CTG)**_**≤50**_ **and *TCF4* (CTG)**_**>50**_ **groups at approximately 3, 6, and 12 months after keratoplasty (Descemet membrane endothelial keratoplasty, DMEK)**. Comparison yielded a *p*‐value of 0.03 for 12 months after surgery CCTs of the left eye; results should be interpreted in the context of a small sample size (TCF4 (CTG)_≤50_ (*n* = 2) and TCF4 (CTG)_>50_ (*n* = 13)). Statistical testing of the patients who had undergone Descemet’s stripping automated endothelial keratoplasty (DSAEK) was not feasible due to the small number of procedures performed in the *TCF4* (CTG)_≤50_ group.

**Table S4**. Exonic and splicing variants with an allele frequency of <0.1 in the Finnish population (gnomAD) observed in FECD candidate genes (*AGBL1*, *COL8A2*, *GRHL2*, *LOXHD1*, *OVOL2*, *SLC4A11*, *TCF4*, *TSPOAP1*, *ZEB1*, and associated loci in *COL18A1*, *HS3ST3B1*, *KANK4*, *LAMA5*, *LAMB1*, *LAMC1*, *LINC00970/ATP1B1*, *PIDD1*, *RORA*, *SSBP3*, and *THSD7A*) in the whole exome data of the 18 patients without the *TCF4* (CTG)_>50_ expansion. Variants are ordered according to their predicted pathogenicity using Varsome germline classification.
