# Supplementary material for: Genetic risk factors in Finnish patients with Fuchs endothelial corneal dystrophy
Source: Acta Ophthalmol. 2026 Apr 20;104(8):873–83. doi: 10.1111/aos.70144 (PMC13751880; doi:10.1111/aos.70144)
Supplement: Supplementary file 1 [file AOS-104-873-s003.pdf]

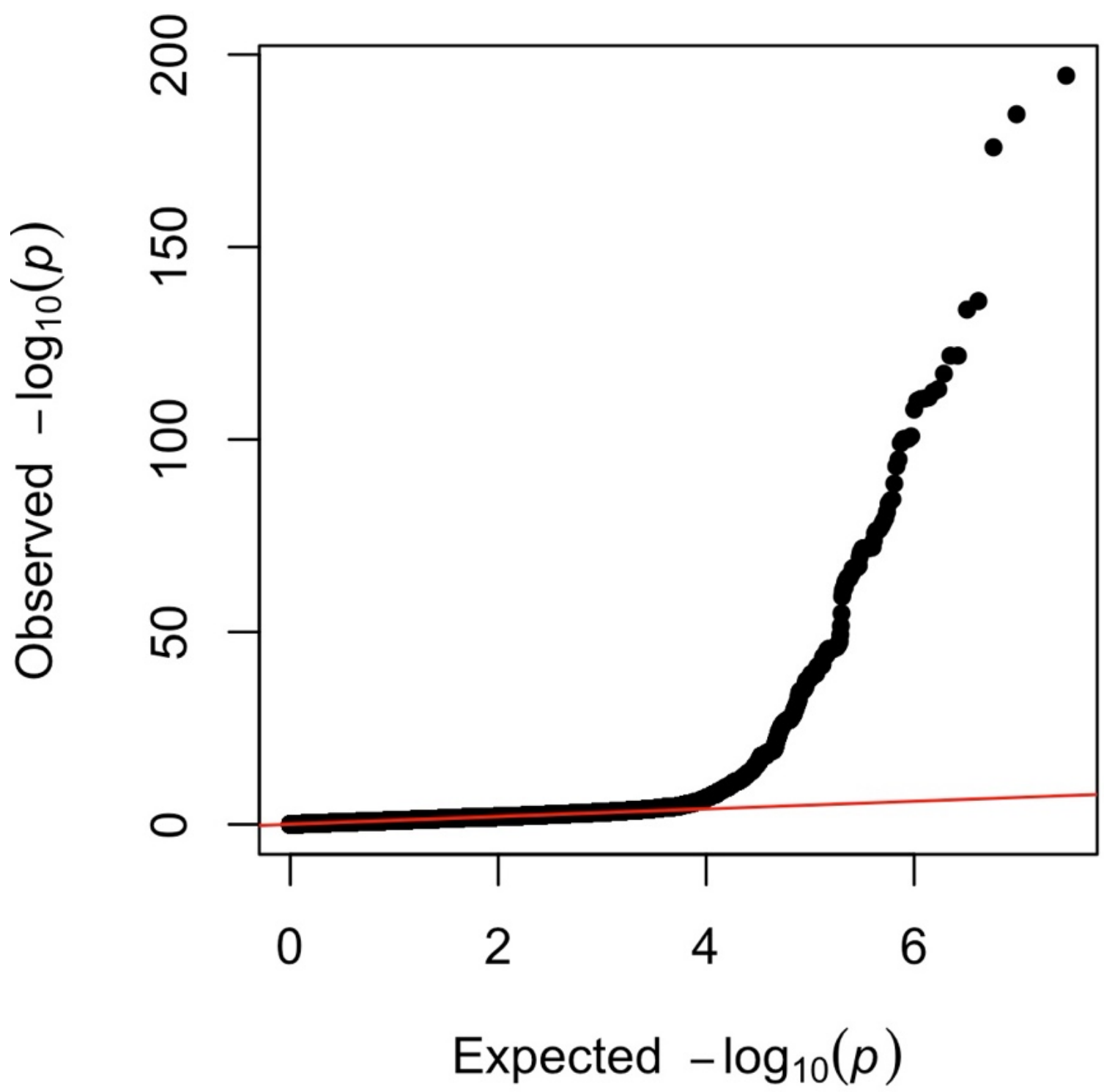

Figure S1. Quantile-quantile plot of p-values from a genome-wide association study of potential FECD in FinnGen. A total of 892 patients and 497,827 controls with potential FECD were identified based on ICD codes, and a case-control genome-wide association study was conducted with Regenie v2.2.4. The expected and observed p-values are plotted on the x and y-axis, respectively. The red line corresponds to a theoretical distribution with no deviation from expected p-values.
